# Concurrent decreases in the prevalence of wheezing and *Ascaris* infection among 5‐year‐old children in rural Bangladesh and their regulatory T cell immunity after the implementation of a national deworming program

**DOI:** 10.1002/iid3.253

**Published:** 2019-06-29

**Authors:** Haruko Takeuchi, Md. Alfazal Khan, Shaikh Meshbahuddin Ahmad, S. M. Tafsir Hasan, Md. Jahangir Alam, Sayaka Takanashi, Samar Kumar Hore, Sultana Yeasmin, Masamine Jimba, Tsutomu Iwata

**Affiliations:** ^1^ Department of Community and Global Health, Graduate School of Medicine The University of Tokyo Tokyo Japan; ^2^ Nutrition and Clinical Services Division International Centre for Diarrhoeal Disease Research Bangladesh; ^3^ Infectious Diseases Division International Centre for Diarrhoeal Disease Research Bangladesh; ^4^ Department of Developmental Medical Sciences, Graduate School of Medicine The University of Tokyo Tokyo Japan; ^5^ Centre for Equity and Health System International Centre for Diarrhoeal Disease Research Bangladesh; ^6^ Department of Education for Childcare, Faculty of Child Studies Tokyo Kasei University Tokyo Japan

**Keywords:** *Ascaris* infection, Bangladesh, childhood wheezing, deworming, Treg cells

## Abstract

**Introduction:**

Epidemiological research on the prevalence of asthma and helminthic infections in various countries has led to the hypothesis that helminthic infections protect against asthma by suppressing the host's immune response. This study was conducted to elucidate whether decreased *Ascaris* infection following a national deworming program was associated with increased recurrent wheezing among rural Bangladeshi children and to test their anti‐inflammatory immunity.

**Methods:**

This nested case‐control study was conducted from December 2015 to October 2016 in the rural service area of the International Centre for Diarrhoeal Disease Research, Bangladesh. Of the 1800 5–year old children randomly selected for the study, informed consent was obtained from the guardians of 1658 children. Data were collected using a semistructured questionnaire adopted from the International Study of Asthma and Allergies in Childhood and blood samples for the analysis of regulatory T (Treg) cell immune responses and the balance between Th1 and Th2 immunity in *Ascaris* infections.

**Results:**

A total of 145 children were found to have wheezing, yielding a prevalence rate of 8.7%, which was significantly lower than the rate found in 2001 (16.2%, *P* < .001); *Ascaris* infection also decreased from 2001 to 2016. The 127 wheezing children who agreed to participate further were compared to 114 randomly selected never‐wheezing children. Wheezing had a significant positive association with antibiotic use, history of pneumonia, parents’ history of asthma, and *Ascaris* infection; children with *Ascaris* infection were twice as likely to have wheezing (adjusted odds ratio = 2.31, *P* = .053). Flow cytometry found no significant differences in the rates of Th1, Th2, and CD4 ^+^CD25 ^+^CD127low cells by the wheezing group.

**Conclusions:**

*Ascaris* infection had a positive rather than a negative association with wheezing and the rates of wheezing and *Ascaris* infections both decreased from 2001 to 2016. These findings undermines the hypothesis that such infections provide protection against asthma.

## 1 INTRODUCTION

The primary risk factors for developing asthma during childhood are atopy and lower respiratory tract infections.^1^, ^2^ The prevalence of asthma has increased rapidly since the 1970s in developed countries, with industrial and urban‐dwelling children experiencing more asthma than those living in rural areas.^3^, ^4^, ^5^, ^6^, ^7^ These findings have led to the hypothesis that helminthic infections might provide protection against asthma by suppressing the host immune response. This relationship remains controversial because the results of multiple epidemiological studies both support and refute the protective effects of helminths on asthma and allergy.^8^, ^9^, ^10^, ^11^

Helminth infections activate regulatory T (Treg) cells and induce the production of interleukin 10 (IL‐10), and thus, play a protective role against asthma and allergy.^8^ Studies have shown that IL‐10 induced in chronic schistosomiasis appeared to be central to suppressing atopy in African children,^8^ and infection with *Schistosoma mansoni* has been associated with a reduced course of asthma.^9^ Nevertheless, this suppressive function remains poorly understood in the context of Ascariasis, in contrast to Schistosomiasis. It appears likely that *Ascaris* infections are associated with increased wheezing. A systematic review and meta‐analysis of 22 studies found an association between *Ascaris* infection and wheezing.^10^ *Ascaris* might induce an inflammatory response in the lungs independent of its effect on IgE production, which should explain the association between geohelminth infection and asthma.^12^ Ardura‐Garcia et al^11^ reported a higher risk of asthma or wheezing associated with an *Ascaris* infestation in a systematic review of studies conducted in Latin America.

Moreover, anti‐Ascaris IgE appears to contribute to the development of wheezing. In our previous study, we reported an association of anti‐Ascaris IgE with wheezing and bronchial hyperreactivity among children in rural Bangladesh, where 72% of them were infected with *Ascaris*.^13^, ^14^ Anti‐Ascaris IgE has also been found to be a risk factor for wheezing and/or atopy among preschool children in Brazil.^15^ In addition, *Ascaris lumbricoides* tropomyosin was found to be crossreactive to mite tropomyosins^16^ and this crossreactivity was found to be partially responsible for the IgE responses to *Ascaris*.^17^ These findings suggest that anti‐*Ascaris* IgE is associated with an increased risk of wheezing, although studies found that *Ascaris* infection was not a risk factor for wheezing. Therefore, the role of anti‐*Ascaris* IgE in the development of asthma remains unclear.

More than 72% of the children in a study we conducted in Matlab, Bangladesh, were infected with *Ascaris* between 2001 and 2005,^13^, ^14^ although it was not a risk factor for wheezing. This prevalence decreased after the introduction of a national deworming program in 2004,^18^ which was developed to administer antihelminthic drugs to children aged 24 to 59 months, with an another program (also initiated in 2004) that delivered the drugs to primary school children. We expected this program to have some impact on the prevalence of wheezing.

Therefore, this epidemiological study aimed to identify children with current wheezing and those who never experienced wheezing, to conduct subsequent testing for helminth infections, and to analyze their lymphocyte subpopulations, particularly their anti‐inflammatory responses, investigating the impact of the national deworming program on wheezing, and examined the Treg immune responses and the balance between Th1 and Th2 immunity during an *Ascaris* infection.

## 2 METHODS

### 2.1 Study design, site, and participants

This nested case‐control study was conducted from December 2015 to October 2016 in rural Bangladesh at Matlab, where International Centre for Diarrhoeal Disease Research, Bangladesh (icddr,b) has been running the Health and Demographic Surveillance System (HDSS) since 1966.^19^ The Matlab HDSS area is located 55 km southeast of Dhaka, the capital city, and encompasses 142 villages with a population of 230 000. The HDSS consists of the icddr,b service area, where the study was conducted and the government service area, which serves as the comparison area. There is a central health facility, Matlab Hospital, which provides free‐of‐charge services to children under 5 years and women of child bearing age residing in the icddr,b service area. Children with suspected pneumonia are referred to Matlab Hospital from the community by the community‐health research workers.

**Figure 1:**
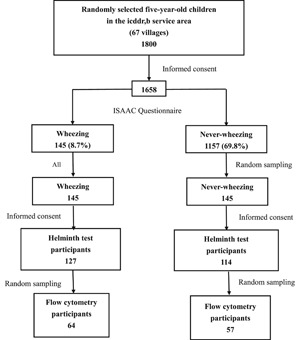
Flowchart of the sampling procedure

A total of 1800 randomly selected children aged 5 years from all 67 villages of the icddr,b service area were asked to participate in the study, of which 1658 (92.1%) children agreed. The International Study of Asthma and Allergies in Childhood questionnaire was used to identify wheezing and never‐wheezing children. We found that 145 children (8.7%) had wheezing in the last 12 months (wheezing), 356 (21.5) had history of wheezing in the first 4 years of life, and 1157 (69.8) had no history of wheezing in their lifetime (never‐wheezing). For this case‐control study, we considered only the wheezing and never‐wheezing children. We invited all the wheezing children (n = 145) and randomly selected equal number of never‐wheezing children. A total of 127 wheezing and 114 never‐wheezing children agreed to participate, and constituted the cases and controls, respectively. For flow cytometry analysis, half of these children (64 cases and 57 controls) were selected randomly (Figure 1).

### 2.2 Field data collection

Trained local field‐research assistants visited the homes of the children, obtained written informed consent from their legal guardians, and collected information using a semistructured, pretested questionnaire adopted from the International Study of Asthma and Allergies in Childhood questionnaire. Wheezing was defined as any episode of wheezing or whistling in the chest in the 12 months preceding the interview. Children who never had any episode of wheezing or whistling in the chest were identified as never‐wheezing children. Information was also collected regarding family history of allergies, socioeconomic status, and environmental factors. Information on participants’ history of pneumonia was collected using the electronic database of Matlab Hospital. The diagnosis of pneumonia was based on the WHO guidelines for management of common illnesses with limited resources.^20^ The children were referred to Matlab Hospital of icddr,b, where a study physician assessed their physical status and confirmed their responses to the questionnaire in face‐to‐face interviews. Between 6:30 and 8:00 am, 7 mL of venous blood and 50 g stool samples were collected from each of the children at the hospital. Data on *Ascaris* infection prevalence and childhood wheezing during the year 2001 were retrieved from our previous study, which was conducted in the same communities and with children in the same age group.^13^

### 2.3 Laboratory testing

Fresh stool specimens were examined for parasites and ova using a direct smear technique on a single stool sample performed by regular laboratory staff of Matlab laboratory of icddr,b and was the same method as used in the 2001 survey. However, this entire process was overseen by a dedicated lab personnel employed by the study. Samples of cases and controls were examined simultaneously. Of the 7 mL of blood collected, 3 mL was taken in a sodium heparin tube (BD Biosciences, San Jose, CA) and transferred at room temperature to the Immunobiology, Nutrition and Toxicology laboratory of icddr,b, as early as possible, preferably before noon for flowcytometry analysis. Of the other 4 mL, which was kept in a cooler, 2 mL of blood was used for complete blood count and the rest was stored as serum for IgE measurement.

The Treg cell population was labelled with a commercially available Human Regulatory T Cell Cocktail kit (CD4‐FITC/CD25‐PECy7/CD127‐AlexFluor647; BD Biosciences, Tokyo, Japan) according to the manufacturer's instructions and the data were collected using a BD FACSAria III flow cytometer (BD Biosciences, Tokyo, Japan). The flow data were analyzed using FlowJo software (FlowJo, San Jose, CA). CD4^+^CD25^int/high^CD127^low^ cells were identified as Treg cells, since they expressed the highest level of Foxp3 and were strongly correlated with naturally occurring Treg cells.^21^

The expression of intracellular Th1 and Th2 cytokines was also determined using flow cytometry as previously described with some modification.^22^ In brief, 500 µL whole‐blood samples were diluted 1:1 with Roswell Park Memorial Institute 1640 in 12 × 75 mm fluorescence‐activated cell sorting (FACS) tubes and activated with phorbol 12‐myristate 13‐acetate (10 ng/mL; Sigma‐Aldrich, Tokyo, Japan) and ionomycin (1 µg/mL; Sigma‐Aldrich, Tokyo, Japan). The cultures were incubated for 4 hours at 37°C and 5% CO_2_ in the presence of brefeldin A (10 µg/mL; Sigma‐Aldrich, Tokyo, Japan). Brefeldin A acts as a transport inhibitor, which prevents cytokine release from cells. Samples incubated with brefeldin A and phosphate‐buffered saline (PBS) were used as unstimulated controls. After this incubation, both the stimulated and PBS control tubes were stained with CD3‐APCCy7 (BioLegend, Tokyo, Japan). Surface staining was followed by red blood cell lysis and cell permeabilization using FACS Lysing Solution (BD Biosciences, Tokyo, Japan) and FACS Permeable Solution (BD Biosciences, Tokyo, Japan), respectively. Intracellular staining of Th1 (IFNγ‐PerCPCy5.5; BioLegend, Tokyo, Japan) and Th2 (IL4‐FITC, IL5‐APC, and IL13‐PerCpCy5.5; BioLegend, Tokyo, Japan) cytokines were performed using matched isotype control tubes. The cells were fixed with 1% paraformaldehyde and maintained at 4°C overnight. Acquisition was performed on the following day with a BD FACSAria III flow cytometer (BD Biosciences, San Jose, CA). Flow data were analyzed using FlowJo software (BD Biosciences, San Jose, CA). Cytokine responses were calculated using the following formula:

Cytokine response=(%activated sample−%activated isotypecontrol)−(%unstimulatedsample−%unstimulatedisotypecontrol)

### 2.4 Statistical analysis

The sample size was determined based on the assumption that at least approximately 16% of the children aged 60 to 71 months would have wheezing.^13^ Given 80% power and a 5% significance level, 209 were required for each group to detect a difference in the serum levels of log anti‐*Ascaris* IgE of 1.8 U_A_/mL (SD, 1.5) and 1.4 U_A_/mL (SD, 1.4) between the wheezing and never‐wheezing children. Thus, we needed to approach 240 wheezing and 240 never‐wheezing children assuming a 15% refusal rate, absences included. To obtain the required number of wheezing children, we needed to approach 1800 individuals with a 20% loss due to absences, refusal, and other reasons.

The data were analyzed using IBM SPSS Statistics version 22 (IBM Japan, Tokyo, Japan). First, the prevalence of wheezing and other asthma symptoms in 2001 and 2016 were compared. An initial exploratory data analysis was conducted to determine the distribution of the outcome variables. After each variable underwent a descriptive analysis between the wheezing and never‐wheezing participants, the continuous variables (eg, height) were compared using a *t*‐test or Mann–Whitney test and the categorical variables were compared using the χ^2^ test. In the nested case‐control study, the outcome variables were Th1, Th2, and Treg frequencies, and were compared among the independent variables for helminth infections with or without the presence of wheezing with a one‐way analysis of variance (ANOVA). Helminth infection was also compared as an outcome variable between the wheezing and never‐wheezing groups. The odds ratios for wheezing with these risk factors were calculated using multiple logistic regression analysis with wheezing status as the dependent variable.

## 3 RESULTS

Informed consent was obtained from the guardians of the 1658 children. Stool samples were collected from 127 wheezing and 114 never‐wheezing children. There was no disagreement in the assessment of wheezing between the field research assistants and the study physicians. Blood samples for flow cytometry analysis were obtained from 64 and 57 children, respectively, and plasma samples from 126 and 110 children, respectively, were stored for future IgE and cytokine quantification. The prevalence of wheezing in the present study was 8.7% in 2016, which was significantly lower than that in 2001 (*P* < .001), when it was 16.2% among 1587 children.^13^ Similarly, the prevalence of *Ascaris* infection decreased significantly among wheezing and never‐wheezing children from 76% and 72% in the year 2001 to 18.9% and 9.7%, respectively.^13^ Among the 127 wheezing and 114 never‐wheezing children, 14 wheezing and two never‐wheezing children were found to have experienced pneumonia.

Table 1 shows the results of the analyses of the factors that were likely to be significantly different between the wheezing and never‐wheezing children. No difference between the groups was found in the percentage of children dewormed in 2016. The factors found to be more prevalent among the wheezing children included the use of antibiotics, parents’ history of asthma, a history of pneumonia, and *Ascaris* infection. Table 2 shows the socioeconomic status of the children in 2001 and 2016, and we observed substantial changes since 2001.^13^ Electricity was available in 65% of households, and the percentage of mothers with no education decreased from 40% to 10%, and monthly income increased three‐fold. According to the adjusted logistic regression, significant risk factors for wheezing included the use of antibiotics, history of pneumonia, mother's asthma, and father's asthma (Table 3). The adjusted OR of *Ascaris* infection was 2.31 (95% confidence interval [CI]: 0.99‐5.38) but it was not significant (*P *= .053). Stool were also examined for eggs or larvae of other helminths and *Trichuris Tritiura* was the only helminth found by the test. We found no significant difference in the prevalence between wheezing and never‐wheezing children (*P* = .187; Table 1). We also tried to detect enteric protozoal cysts and found *Blastocystis hominis* cysts in four children (one in wheezing and three in never‐wheezing children) and *Giardia lamblia* cysts in seven children (four in wheezing and three in never‐wheezing children). The difference in the prevalence of both cysts between wheezing and never‐wheezing group was not statistically significant by the exact test (*P* = 1.000).

**Table 1:** Characteristics of the wheezing and never‐wheezing children

		Wheezing	Never‐wheezing	
		(n = 127)	(n = 114)	*P*
Sex	F/M	63/63	65/45	.162
Deworming^a^	Yes	126 (100%)	108 (98.2%)	.216
Use of antibiotics^b^	Yes	121 (96.8%)	98 (89.1%)	.035
Contact with livestock^c^	Yes	99 (78.6%)	76 (69.1%)	.097
Contact with cats^d^	Yes	8 (6.4%)	7 (6.3%)	1.000
Household smoking	Yes	51 (40.5%)	36 (32.7%)	.217
Dry leaves for fuel	Yes	50 (39.7%)	32 (29.1%)	.088
Mother has asthma	Yes	22 (17.5%)	5 (4.5%)	.005
Rhinitis	Yes	33 (26.4%)	22 (20.0)	.248
Father has asthma	Yes	16 (12.8%)	5 (4.5%)	.038
Rhinitis	Yes	31 (19.0%)	19 (17.3%)	.201
Pneumonia ever^e^	Yes	14 (11.1)	2 (1.8)	.004
*Ascaris* infection	Yes	24 (18.9%)	11 (9.7%)	.047
*Trichuris* infection	Yes	28 (22.2%)	17 (15.5%)	.187

^a^ Has the child received any deworming drug?

^b^ Did you give antibiotic during the first 12 mo of your child?

^c^ Did the child have regular (at least once a week) contact with farm animals or livestock during the first year of life? (eg, cattle, goats, sheep, poultry, etc)

^d^ Did you have a cat in your home during the first year of your child's life?

^e^ Has the child ever had history of pneumonia? History of pneumonia was drawn from the clinical chart of Matlab Hospital and had been diagnosed based on WHO criteria.^20^

**Table 2:** Comparison of socioeconomic development in Bangladesh in 2001 and 2016

			2001	2016
Year			(n = 1587)	(n = 1658)
Height	cm	Mean	103.5	107.8
Weight	kg	Mean	14.8	16.5
Electricity	Yes		423 (26.7%)	1076 (64.9%)
Father's education	Higher		141 (8.9%)	295 (17.8%)
	Middle		438 (27.6%)	585 (35.4%)
	Primary		470 (29.6%)	482 (29.1%)
	No education		538 (33.9%)	292 (17.7%)
Mother's education	Higher		44 (2.8%)	159 (9.6%)
	Middle		335 (21.1%)	950 (57.4%)
	Primary		569 (35.8%)	379 (23.0%)
	No education		639 (40.3%)	166 (10.0%)
Monthly income	BDT^a^	Mean	4589	16750

^a^ Bangladesh Taka.

**Table 3:** Adjusted odds ratios for wheezing

			Crude OR (95% CI)		*P*	Adjusted OR^a^ (95% CI)		*P*
Sex	M/F^b^	63/63	1.44	(0.86‐2.42)	.163	1.18	(0.67‐2.09)	.582
Use of antibiotics	Yes/No	121/6	3.70	(1.16‐11.84)	.027	3.94	(1.09‐14.24)	.036
Pneumonia history	Yes/No	14/113	6.75	(1.50‐30.40)	.013	5.71	(1.18‐26.24)	.030
*Ascaris* infection	Yes/No	24/103	2.12	(0.99‐4.55)	.055	2.31	(0.99‐5.38)	.053
Mother has asthma	Yes/No	22/105	4.49	(1.64‐12.30)	.004	4.50	(1.56‐13.00)	.005
Rhinitis	Yes/No	33/94	1.44	(0.78‐2.65)	.249	1.23	(0.59‐2.57)	.587
Father has asthma	Yes/No	16/111	3.08	(1.09‐8.72)	.034	3.02	(1.01‐9.05)	.040
Rhinitis	Yes/No	31/96	1.58	(0.83‐3.00)	.161	1.21	(0.56‐2.61)	.595
Wood for fuel	Yes/No	50/77	0.99	(0.54‐1.80)	.975	0.77	(0.40‐1.51)	.452
Contact with livestock	Yes/No	99/28	1.64	(0.91‐2.95)	.098	1.28	(0.67‐2.45)	.082

Abbreviations: CI, confidence interval; OR, odds ratio.

^a^ Adjusted for each other.

^b^ Female is the reference category.

Table 4 shows the characteristics of the children who underwent flow cytometry by presence of wheezing and *Ascaris* infection. We found a marked difference in the number of the participants between those with and without *Ascaris* infection. When we compared the rate of Th1, Th2, and CD4^+^CD25^+^CD127^low^ cells by presence of wheezing, we found no significant difference between wheezing and never‐wheezing children (Table 5). No significant difference was found among the four groups of wheezing and never‐wheezing children with or without an *Ascaris* infection using one‐way ANOVA because of the small number of the children with *Ascaris* infection (Table 5).

**Table 4:** Characteristics of the children who underwent flow cytometry was done by presence of wheezing and *Ascaris* infection

	Wheezing children		Never‐wheezing children		
	*Ascaris* infection	No *Ascaris* infection	*Ascaris* infection	No *Ascaris* infection	*P*
N =	(n = 7)	(n = 57)	(n = 3)	(n = 54)	
Female/Male	1 (14%)/6 (86%)	30 (53%)/27 (47%)	1 (33%)/2 (67%)	27 (50%)/27 (50%)	.262
Pneumonia (+)	3 (43%)/4 (57%)	6 (11%)/51 (90%)	0 (0%)/3 (100%)	0 (0%)/54 (100%)	<.001
Mother's asthma	0 (0%)/7 (100%)	8 (14%)/49 (86%)	0 (0%)/3 (100%)	4 (7%)/50 (93%)	.464
Father's asthma	0 (0%)/7 (100%)	10 (18%)/47 (83%)	0 (0%)/3 (100%)	1 (2%)/53 (98%)	.025
Livestock exposure	7 (100%)/0 (0%)	49 (86%)/8 (14%)	3 (100%)/0 (0%)	36 (67%)/18 (33%)	.028
Antibiotic use	7 (100%)/0 (0%)	56 (100%)/0 (0%)	3 (100%)/0 (0%)	47 (87%)/7 (13%)	.028
Smoking	2 (29%)/5 (71%)	30 (53%)/27 (47%)	0 (0%)/3 (100%)	25 (46%)/29 (53%)	.193

**Table 5:** Median response of Th1, Th2, and Treg cells with interquartile ranges

	Wheezing children		Never‐wheezing children		
	*Ascaris* infection	No *Ascaris* infection	*Ascaris* infection	No *Ascaris* infection	*P*
%	(n = 7)	(n = 57)	(n = 3)	(n = 54)	
CD4^+^ IFN γ	8.5 (2.2‐14.7)	9.2 (0.6‐17.7)	7.5 (7.5‐7.5)	9.8 (2.9‐16.6)	.858
CD4^+^ IL‐12	0.5 (0.3‐1.0)	0.5 (0.3‐0.7)	0.5 (0.5‐0.5)	0.5 (0.2‐0.7)	.937
CD4^+^ IL‐4	3.7 (0.8‐6.6)	4.8 (0‐8.3)	1.2 (1.2‐1.2)	3.5 (1.2‐6.7)	.144
CD4^+^ IL‐5	0.8 (0‐1.6)	1.0 (0‐2.4)	0.7 (0.7‐0.7)	0.9 (0.0‐1.7)	.756
CD4^+^ IL‐13	3.3 (0.5‐6.0)	2.0 (0‐6.0)	1.0 (1.0‐1.0)	3.0 (0‐6.3)	.055
Treg^a^	6.7 (4.8‐8.6)	7.2 (4.9‐9.6)	7.0 (7.0‐7.0)	7.4 (5.3‐9.5)	.765

Abbreviations: IFN, interferon; IL, interleukin; Treg, regulatory T.

^a^ CD4^+^CD25^int/high^CD127^low^ cells.

## 4 DISCUSSION

The prevalence of wheezing was significantly lower and *Ascaris* infections had decreased in 2016 compared to 2001. In addition, wheezing children had a significantly higher rate of *Ascaris* infection compared to never‐wheezing children, although *Ascaris* infection was not a risk factor for wheezing. Despite the significant decrease in *Ascaris* infections, which are thought to increase asthma, wheezing prevalence did not increase. Furthermore, no significant difference was found in the percentage of CD4^+^CD25^+^CD127^low^ cells between the wheezing and never‐wheezing children without an *Ascaris* infection (Table 4).

The present study revealed a remarkable decline in the prevalence of wheezing among 5–year old children. Previous studies have reported that the prevalence of asthma in Bangladesh has increased in recent years. A nationwide cross‐sectional survey in 1999 reported an asthma prevalence of 7.3% among children aged 5 to 14 years.^23^ A subsequent study conducted in 2000 among school children aged 6 to 7 years reported a wheezing rate of 9.1%.^24^ In one of our studies conducted later, we reported that the prevalence of current wheezing was 16% in 2001 among children aged 5 to 6 years in a rural area.^25^ Furthermore, a 2008 study reported a 19.7% prevalence of current wheezing in the same area among 5–year old children.^18^ In contrast to these trends, the present study found a significant decrease in the prevalence of wheezing. During the period between 2001 and 2016, we found substantial changes in the socioeconomic status. Electricity was available in 65% of households, mothers with no education decreased from 40% to 10%, and monthly income increased three‐fold between 2001 and 2016. As bronchial asthma is assumed to increase in conjunction with regional development, we speculate there must be some other unique reason for the decrease in wheezing.

The present study found a decrease in the burden of helminth infections, with an *Ascaris* infection rate of 10.2%, which was higher among wheezing children. The adjusted odds ratio of an *Ascaris* infection for wheezing was 2.31 (*P* = .053), which was not significant. The prevalence of *Ascaris* eggs in the stool was higher than 72% among 5–year old rural children in 2001 and 2005.^13^, ^14^ Contrary to the general assumption that the prevalence of asthma increases with a decline in the burden of helminth infections, our study found a concurrent decrease in the prevalence of wheezing and *Ascaris* infection, which is inconsistent with the notion that a helminth infection is a protective factor for asthma. Moreover, this result did not contradict our previous finding in 2001, that *Ascaris* infection was not a risk factor for wheezing, although anti‐*Ascaris* IgE was a risk factor for wheezing. In other words, *Ascaris* infection was not an increasing risk factor in the present study, just as it was not a risk factor for wheezing in the 2001 study.

National deworming program was initiated in Bangladesh in 2004 to administer antihelminthic drugs twice a year to children aged 24 to 59 months along with an additional program for primary school children. It is assumed that the program had a substantial impact on the infectious burden of helminths among children. The prevalence of *Ascaris* infections in Bangladesh supports this assumption as the *Ascaris* worm burden among adults in 1998 was between 64% and 95%^26^ and decreased to 17.4% in 2008.^15^ In 2012, the *Ascaris* egg prevalence was 13% among children aged 1 to 4 years and 14% among those aged 4 to 14 years.^27^ Although our study could not show that *Ascaris* infection was a greater risk factor for wheezing, the decrease in *Ascaris* infections did not increase wheezing in children, either. Thus we infer helminth infection does not protect against asthma and allergy.

We found no significant difference in the percentage of CD4^+^CD25^+^CD127^low^ cells between wheezing and never‐wheezing children without an *Ascaris* infection (Table 4). Circulating Treg cells were not involved in the suppression of wheezing, although they might have been present locally in the airway. Neither Th2 nor Th1 cells were associated with increased wheezing. We attempted to elucidate the potential for *Ascaris* to induce Treg immunity because helminth infections have been shown to induce the downregulation of the host immune response via Treg cell activation and IL‐10 production, as a mechanism employed by helminths to escape host immune surveillance and promote their survival in the host. Indeed, *Schistosoma*‐associated CD4^+^CD25^high^FOXP3^+^ Tregs have been found to exert a suppressive effect on both T cell proliferation and cytokine production.^28^ Such regulatory responses are thought to function to suppress both Th1‐ and Th2‐mediated inflammation in the host, and thus, the development of asthma and allergy.

Initially, we estimated the results of Th1, Th2, and Treg responses as follows: Th2 responses would be the highest among wheezing children with *Ascaris* infection and lowest among never‐wheezing children without *Ascaris* infection. The responses would be higher among children with *Ascaris* infection than children without *Ascaris* infection both in wheezing and never‐wheezing children. Th1 responses would not be so much different throughout the four groups. Treg responses would be slightly elevated among children with *Ascaris* infection. This study found that Th1 and Treg responses were not different from our estimates. However, against our expectations, Th2 response presented as CD4^+^IL‐13 was slightly higher in never‐wheezing children without *Ascaris* infection than in wheezing children without *Ascaris* infection but without significance. As little is known about the suppressive effect of Treg cells and IL‐10 in Ascariasis, which may not necessarily function in the same manner as in Schistosomiasis, we should verify the contribution of these cells to wheezing in the future studies.

We could not compare Treg cells between children with and without *Ascaris* infection, either, for the reason that the sample size of the children with *Ascaris* infection was too small to analyze because the number of the uninfected children who underwent flow cytometry analysis was much lower than we initially planned. The most dominant reason for the reduction in the number of the participants in the cell of wheezing children with *Ascaris* infection was the reduction of wheezing prevalence down to 8.7%. Since the wheezing prevalence had been increasing for these 20 years, we estimated the prevalence to be at least more than 16% and expected the sample size for flowcytometry for the cell of wheezing children with *Ascaris* infection to be at least 20. Thus, together with the lower prevalence of *Ascaris* infection than our expectation, the number of the children in the cell resulted in much smaller than our expectation.

The initial purpose of the present study was to demonstrate that anti‐*Ascaris* IgE was a risk factor for wheezing among 5–year old rural Bangladeshi children. We wanted to confirm our study result in 2001 that anti‐*Ascaris* IgE was a risk factor for wheezing among 5–year old rural Bangladeshi children. Thus, we estimated the sample size based on the serum level of anti‐*Ascaris* IgE. Since anti‐*Ascaris* IgE will not be elevated without *Ascaris* infection, we think that *Ascaris* infection might have any association with wheezing. We could not measure the total IgE, anti‐*Dermatophagoides pteronyssinus* (Dp) IgE, anti‐*Ascaris* IgE or Th cytokines in the present study, although we previously demonstrated that elevated levels of serum anti‐*Ascaris* IgE were associated with wheezing and BHR in children in rural Bangladesh, where up to 72% of children were infected with *Ascaris.* 13, 14 This finding was later supported by a subsequent study conducted in the same region of Bangladesh, in 2008, when the infection prevalence was 17.4%. That study reported that anti‐*Ascaris* IgE was associated with an increased risk of ever having asthma among 5–year old children.^18^ Nakazawa et al^29^ reported that *Ascaris lumbricoides* antigens induced the production of antibodies crossreactive to mite antigens from *Dermatophagoides farina*, indicating that mast cell‐bound anti‐*Ascaris* IgE on the airway surface produced in the infected individual might crossreact with the inhaled *Dermatophagoides* antigen present in the environment. Therefore, the coexposure to mite and *Ascaris* antigens might increase the risk of wheezing.^30^ Although we could not measure the total IgE, anti‐Dp IgE, anti‐*Ascaris* IgE, or Th cytokines, it is natural that anti‐*Ascaris* IgE increases in those individuals with current or past *Ascaris* infections. Thus, the notion that childhood wheezing in rural Bangladesh might be attributable to *Ascaris* infection is not incongruous.

It is well known that *Ascaris* migrates through the lungs during maturation and causes wheezing known as Loffler syndrome. Calvert et al reported that *Ascaris* might induce an inflammatory response in the lungs independent of its effect on IgE production, which might explain some of the contradictory findings reported in studies examining the association between geohelminth infection, atopy, and asthma.^11^ Therefore, the type of wheezing we observed might have been caused by pulmonary eosinophilic inflammation independent of IgE. For the time being, we are waiting for the financial support for the measurement of IgEs to obtain solution for this question. It is a new finding that wheezing prevalence decreased despite decreased *Ascaris* infection, which we consider merits publication.

A limitation of this study is that the children were grouped according to the presence of wheezing, which led to an insufficient number of participants with *Ascaris* infection. Thus we think that we should have divided the group by *Ascaris* infection. We might have found additional evidence regarding the contribution of *Ascaris* to wheezing if we had used this alternative grouping method. The second limitation is that we could not include anti‐*Ascaris* IgE, which was an important risk factor for recurrent wheezing in 2001, in the present analysis. Moreover, the sample‐size calculation was based on the serum levels of anti‐*Ascaris* IgE, not the prevalence of *Ascaris* infection. We plan to publish this study because we consider it important to report the substantial impact of the deworming program to the public in Bangladesh before we obtain the financial aid to test measures of it. The third limitation is that we did not take into account the effects of the improvements in treatment strategies. Given the socioeconomic development during this period, we might expect increased drug compliance that could have helped reduce recurrent wheezing.

Childhood wheezing in rural Bangladesh decreased simultaneously with the decrease in *Ascaris* infection. In other words, the decrease in *Ascaris* infection did not increase the prevalence of wheezing nor increase asthma. The underlying mechanism for such wheezing still remains unclear. We speculate that *Ascaris* might contribute to childhood wheezing through the complex interplay between regulatory, acquired, and innate immunity. Therefore, the role of Treg cells and anti‐*Ascaris* IgE in *Ascaris* infection in childhood wheezing merits further exploration.

## ETHICS, CONSENT, AND PERMISSIONS

The study protocol PR‐15054 was approved by the Ethical Review Committee of icddr,b. The Ethics Committee of Tokyo Kasei University approved the study protocol (Sayama H27‐09), and protocol 11018 was approved by the Ethics Committee of The University of Tokyo. This study involved human subjects, and therefore, followed the ethical principles of the Declaration of Helsinki. Written informed consent was obtained from the legal guardians of all the participants.
