# Correction to “Concurrent decreases in the prevalence of wheezing and *Ascaris* infection among 5‐year‐old children in rural Bangladesh and their regulatory T cell immunity after the implementation of a national deworming program”

**DOI:** 10.1002/iid3.951

**Published:** 2023-12-20

In Takeuchi et al.,^1^ the Acknowledgements and Funding statements are incorrect and should be corrected.

The incorrect Acknowledgements read:
